# AI-Assisted Response Surface Methodology for Growth Optimization and Industrial Applicability Evaluation of the Diatom *Gedaniella flavovirens* GFTA21

**DOI:** 10.3390/bioengineering12111277

**Published:** 2025-11-20

**Authors:** Eun Song Kim, Soo Jeong Lee, Jung A Lee, Sung Min An, Hyun-Ju Hwang, Bum Soo Park, Hae-Won Lee, Cheol-Ho Pan, Daekyung Kim, Kichul Cho

**Affiliations:** 1 Department of Biological Application and Technology, National Marine Biodiversity Institute of Korea, Seocheon 33662, Republic of Korea; 2 Department of Environmental Science, Hanyang University, Seoul 04763, Republic of Korea; 3 Department of Food Bioengineering, Jeju National University, Jeju 63243, Republic of Korea; 4 Microalgae Ask Us Co., Ltd., Gangneung 25441, Republic of Korea; 5 Daegu Center, Korea Basic Science Institute (KBSI), Daegu 41566, Republic of Korea

**Keywords:** artificial intelligence, ChatGPT, *Gedaniella flavovirens*, industrial potential, optimization, response surface methodology

## Abstract

Although AI-mediated approaches provide promising support for bioengineering using training datasets, their application in microalgal research remains limited. In this study, ChatGPT-4.0, an easily accessible AI model, was employed to optimize culture conditions and evaluate the industrial potential of the isolated diatom *Gedaniella flavovirens*. Culture optimization was conducted using response surface methodology, in which pH, temperature, and salinity were selected as independent variables. ChatGPT assisted in determining the design and suggested a face-centered central composite design. The optimal conditions for biomass production were determined to be pH 8.30, 23 °C, and 34.24 psu. Analysis of variance revealed significant quadratic effects (*p* < 0.05), indicating curvature in the response surface. Fatty acid profiling showed high levels of palmitoleic acid, palmitic acid, and eicosapentaenoic acid. Pigment analysis further indicated a high abundance of fucoxanthin, diadinoxanthin, and diatoxanthin. Based on the analyzed compounds, ChatGPT suggested potential applications of the algal strain across various industrial sectors. The most relevant application was identified as aquafeed, as the strain contains metabolites known to enhance pigmentation, growth, and immune responses in aquaculture species. Overall, this study demonstrates ChatGPT-mediated bioengineering as a practical strategy for optimizing culture conditions and evaluating the industrial potential of novel microalgal strains.

## 1. Introduction

Diatoms, a major group of microalgae with distinctive silica cell walls (frustules), contribute to more than 20% of global carbon fixation and play a significant role in marine ecosystems [1,2]. Their high production of valuable cellular compounds, such as lipids and carotenoids, makes them suitable as bioresource for industrial applications [3,4,5]. For instance, the biomass of the diatom *Phaeodactylum tricornutum* Bohlin, 1897 has been successfully used as a functional ingredient to enhance external pigmentation in gilthead seabream (*Sparus aurata* Linnaeus, 1758), and as a source of nutraceuticals and cosmetics owing to its valuable cellular components [6,7]. Furthermore, Araújo et al. [8] demonstrated that a mixed microalgal diet comprising the cryptophyte *Rhodomonas marina* (Dujardin) Lemmermann, 1899 and the diatom *Skeletonema costatum* (Greville) Cleve, 1873 can be effectively used to support the cultivation of sea urchin *Paracentrotus lividus* (Lamarck, 1816) larvae.

Despite the potential of diatoms, only a limited number of species, such as *P*. *tricornutum*, *S. costatum*, *Odontella aurita* (Lyngbye) C. Agardh, 1832, and *Thalassiosira pseudonana* Hasle & Heimdal, 1970, have been extensively investigated for industrial applications because of their high biomass productivity, well-characterized genomes, and ease of cultivation [9,10,11]. Therefore, it is important to explore other diatom species with promising growth performance and valuable cellular components.

The rapid advancement of artificial intelligence (AI) and its integration into experimental research have become increasingly important [12,13,14]. In recent years, AI has been actively applied in medicine and biotechnology, including clinical named entity recognition, biomacromolecule analysis, drug development, and computational biology [14,15].

ChatGPT, an easily accessible and large transformer-based language model, employs deep learning to process vast amounts of specialized knowledge, thereby enabling logical reasoning and inference generation in biological research [16]. Recent studies have demonstrated its effectiveness in extracting information from biological literature, particularly in microbial performance assessments [17]. Furthermore, it has been applied to predict research trends in synthetic biology [18]. Although AI tools have been widely adopted across various biotechnological disciplines, their application in algal biotechnology remains largely unexplored despite the growing demand for data-driven approaches.

*Gedaniella flavovirens* (H.Takano) Chunlian Li, Witkowski & Ashworth 2018 (Order Fragilariales) is a marine diatom found in brackish environments and serves as an ecological indicator for pollution assessments [19,20,21]. This species has shown remarkable silica removal efficiency, effectively reducing dissolved inorganic silica, phosphate, and ammonia, while enhancing biomass production [20,22]. Furthermore, recent studies have identified *G*. *flavovirens* as a promising source of nano-silica for the fabrication of silver–silica hybrid nanoparticles, offering a potential solution to the stability limitations of conventional silver nanoparticles [23].

Although the potential of *G. flavovirens* has been recognized, information on its cellular composition and broader biotechnological applications remains limited. Moreover, conventional manual optimization of culture conditions and evaluation of industrial potential based on cellular characteristics and bioactive compounds are often time-consuming and inefficient.

We hypothesized that a ChatGPT-assisted approach could provide a practical strategy for optimizing culture conditions through response surface methodology (RSM) and predicting industrial applicability based on cellular composition data. ChatGPT was employed as a text-mining and reasoning aid to select an appropriate RSM design and validate factor ranges by rapidly integrating information from relevant literature. This process, which is typically labor-intensive and subjective when performed using conventional RSM tools, was significantly accelerated by ChatGPT’s contextual synthesis capability, thereby enhancing both the efficiency and reproducibility of the design phase. This study demonstrates the practical application of ChatGPT-4.0 for accelerating RSM-based optimization and assessing the industrial potential of the newly isolated diatom *Gedaniella flavovirens* GFTA21.

## 2. Materials and Methods

### 2.1. Axenicity and Cultivation of G. flavovirens

The diatom *G. flavovirens* GFTA21 (Resource ID: MABIK LP00000217) was obtained from the Marine Microalgae Biobank (https://www.mbris.kr/pub/main/publicMainPage.do accessed on 4 January 2024) at the National Marine Biodiversity Institute of Korea. To establish axenicity, the strain was first cultured in Si-containing F/2 medium (G9903, Sigma Aldrich Co., St. Louis, MO, USA) supplemented with 1.5% agar using the spreading and streaking method. Afterward, algal colonies were then repeatedly sub-cultured on fresh solid F/2 medium. Axenicity was confirmed by inoculating colonies onto Marine Agar (MA, 1.5%) and Luria–Bertani (LB, 1.5%) bacterial culture media and verified by PCR using universal 16S rRNA bacterial primers 27F and 1492R [24].

The axenic strain was transferred to a 50 mL T-flask (SPL, Pocheon, South Korea) containing 25 mL of liquid F/2 medium and incubated in a shaking growth chamber (HB-201MS, Hanbaek Scientific Technology Co., Bucheon, South Korea) under controlled conditions: 20 °C, continuous illumination at 60 μmol/m^2^/s from cool-white, fluorescent light. The resulting brown algal cultures were used in subsequent experiments.

### 2.2. Design of Experiments (DoE) Through Interaction with ChatGPT-4.0

The ChatGPT-4.0 model (OpenAI, San Francisco, CA, USA; 22 August 2024 version) was employed in this study. To ensure the integrity of the responses and to evaluate whether the AI model itself could be effectively used to predict the industrial potential of microalgae, no plugins or external databases were utilized. All ChatGPT-4.0-generated outputs were derived exclusively from the model’s internal knowledge base. Each query was conducted in a new session to prevent prior interactions from influencing subsequent results. Since ChatGPT-4.0 can generate slightly varied responses across different instances, each query was posed only once, and a single response was selected for analysis. Prompts were structured to include a concise summary of biological ranges, a clear question for model recommendation, and coded levels for independent variables. Selection of ChatGPT outputs followed criteria for scientific plausibility, physiological consistency, and literature agreement.

### 2.3. Estimation of Optimal Culture Condition Based on RSM

RSM was conducted using Minitab statistical software (version 18.1; Minitab LLC., State College, PA, USA). A face-centered central composite design (FCCCD), as suggested by ChatGPT, was applied to evaluate the effects of pH, temperature, and salinity on algal biomass production (g/L) and to determine the optimal growth conditions. The range of values for temperature (10–30 °C), salinity (10–60 psu), and pH (6–10) was determined and validated using ChatGPT-4.0 (Table 1). A FCCCD was used, comprising 40 experimental runs including factorial, axial, and center points (Table 2). Randomized cultivation experiments were designed on RSM’s DoE.

The strain was cultivated in 25 mL of F/2 medium with Si in a T-flask (SPL, Pocheon, South Korea) with an initial cell concentration of 10^5^ cells/mL of *G*. *flavovirens* GFTA21. Incubation was carried out in a multi-chamber shaking incubator (HB-201MS, Hanbaek Scientific Technology, Bucheon, South Korea) at 135 rpm under 60 μmol/m^2^/s illumination from cool-white, fluorescent light. After 7 days, corresponding to the late exponential growth phase, biomass (g/L) was measured.

Algal biomass (g/L) was quantified gravimetrically. In brief, a 10 mL culture aliquot was centrifuged at 12,000× *g* for 2 min, freeze-dried overnight using a freeze dryer (FreezZone 4.5 L, Labconco Corp., Kansas City, MO, USA), and the dry pellet was weighed with a microbalance.

The relationship between the response variable *Y* and the independent variables—pH (*X*_1_), temperature (*X*_2_), and salinity (*X*_3_)—was modeled using the following quadratic Equation (1):

*Y* = *β*_0_ + *β*_1_·*X*_1_ + *β*_2_·*X*_2_ + *β*_3_·*X*_3_ + *β*_1__1_·*X*_1_^2^ + *β*_22_·*X*_2_^2^ + *β*_3__3_·*X*_3_^2^ + *β*_1__2_·*X*_1_·*X*_2_ + *β*_1__3_·*X*_1_·*X*_3_ + *β*_2__3_·*X*_2_·*X*_3_ (1)

Where *Y* is the response variable (outcome of interest), and *X*_1_, *X*_2_, and *X*_3_ correspond to pH, temperature, and salinity, respectively. *β*_0_ is the intercept, while *β*_1_, *β*_2_, and *β*_3_ are the linear coefficients for pH, temperature, and salinity, Additionally, *β*_11_, *β*_22_, and *β*_33_ are the quadratic coefficients, and *β*_12_, *β*_13_, and *β*_23_ represent the interaction coefficients describing the combined effects of these factors on *Y*.

### 2.4. Photosynthetic Pigment Analysis

The photosynthetic pigment content of the diatom was analyzed at the Korea Basic Science Institute (KBSI). To extract the photosynthetic pigments, 6 mL of algal culture was centrifuged at 12,000× *g* for 2 min. The pellet was resuspended in 1 mL methanol (>99.9%; JT Baker, Loughborough, UK) for extraction. The extract was filtered through a 0.22 μm polytetrafluoroethylene syringe filter (Whatman, Clifton, NJ, USA) before high-performance liquid chromatography (HPLC) analysis.

HPLC was performed using a diode array detector (DAD) system (1260 Infinity, Agilent, Germany) with a Spherisorb 5.0 μm ODS1 (4.6 × 250 mm) column (Waters, Milford, MA, USA). The mobile-phase system was prepared as per Baek et al. [25]: solvent A (14% of 0.1 M Tris-HCl, pH 8.0; 84% acetonitrile; 2% methanol, v/v) and solvent B (32% acetonitrile; 68% methanol, v/v). The column temperature was maintained at 40 °C, with a 1.2 mL/min flow rate under the following gradient conditions: 100% solvent A from 0 to 15 min, followed by 100% solvent B from 15 to 19 min, and the post-run was performed for 6 min using solvent A.

Pigments were detected at 445 nm and 670 nm for carotenoids and chlorophylls, respectively. Identification was performed by comparing HPLC retention times and absorption spectra against authentic chlorophyll and carotenoid standards (DHI, Hørsholm, Denmark).

### 2.5. Fatty Acid Composition Analysis

Lipid extraction was performed following Hara and Radin [26]. Extracted lipids were subjected to transesterification by heating for 5 min with a mixed liquid catalyst (methanol, 0.5% (v/v) sodium methoxide, and 2.5% (v/v) sulfuric acid). The resulting fatty acids were analyzed using a 7890A gas chromatograph coupled to a 5975C mass-selective detector (Agilent, Santa Clara, CA, USA). For chromatographic separation, a DB-FFAP column was utilized (30 m length, 250 μm internal diameter, 0.25 μm film thickness; Agilent). The gas chromatography oven was programmed to start at 50 °C, holding for 1 min before ramping to 200 °C at 10 °C/min for 30 min. The temperature was then increased to 240 °C at the same rate and maintained for 20 min. Each sample (1 μL) was injected with a split ratio of 20:1, using helium as the carrier gas at a steady flow rate of 1 mL/min. The mass spectrometer operated in electron-impact mode at 70 eV, with the injector and ion source temperatures set at 250 °C and 230 °C, respectively. Data acquisition covered a mass range of 50–550 m/z, and compounds were identified by comparing mass spectra with Wiley/NBS library references. Matches with similarity scores > 90% were considered reliable.

### 2.6. Statistical Analysis

DoE, Analysis of variance (ANOVA), and RSM modeling were conducted using Minitab statistical software (version 18.1; Minitab LLC, State College, PA, USA). Optimal culture conditions were determined using Minitab’s Response Optimizer. Fatty acid and photosynthetic pigment compositions were analyzed in triplicate, and the results are presented as mean ± standard deviation (SD).

## 3. Results and Discussion

### 3.1. Optimization Results via ChatGPT-4.0-Assisted RSM Analysis

**Figure 1:**
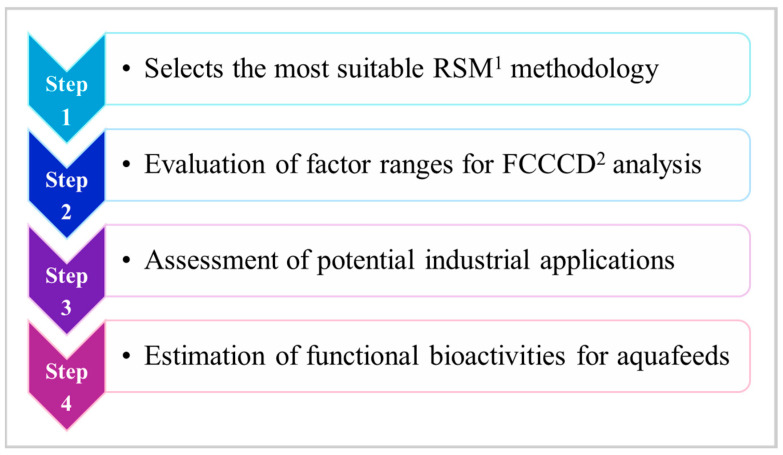
Schematic representation of the four ChatGPT-4-supported analytical steps applied to *Gedaniella flavovirens* GFTA21. ^1^ Response Surface Methodology (RSM); ^2^ Face-Centered Central Composite Design (FCCCD).

As shown in Figure 1, the overall workflow consisted of four steps. The detailed structure and evaluation criteria of the ChatGPT prompts are described in Section 2.2. A representative example of the user–ChatGPT interaction used to determine the experimental design is presented below. ChatGPT-4.0 assistance was incorporated to support (i) the selection of the most suitable RSM, (ii) the evaluation of factor ranges for FCCCD analysis, (iii) the assessment of potential industrial applications, and (iv) the estimation of functional bioactivities for aquafeeds (Figure 1). As a first step, the appropriate RSM between Central Composite Design (CCD) and Box–Behnken Design (BBD) was determined through communication with AI. Since the optimal culture conditions of *G. flavovirens* have not been previously reported, the biological range documented for the related species *Gedaniella panicellus* Chunlian Li, S.X.Yu & Witkowski 2018 by Cho et al. [27] (pH 6–10, 10–30 °C, 10–60 psu) was provisionally adopted for *G. flavovirens* GFTA21. To systematically identify an RSM design appropriate for this biological range, the following interaction demonstrates how prompt-based reasoning was applied to guide design selection.

User prompt: Recent work on *G*. *panicellus* [27] shows that cultures remain viable across pH 6–10, 10–30 °C and 10–60 psu, although biomass declines toward some edge combinations. Because no preliminary culture data exist for *G*. *flavovirens* GFTA21, we wish to model quadratic and two-factor interaction effects within this biological range using response-surface methodology. Please code the factors at −1 (pH 6, 10 °C, 10 psu), 0 (pH 8, 20 °C, 35 psu) and +1 (pH 10, 30 °C, 60 psu), a face-centered CCD, or a BBD best meets these constraints. Please answer concisely.

ChatGPT-4.0 response: A face-centered central composite design (FCCCD, α = 1) is the most appropriate choice given the stated constraints. It allows accurate estimation of quadratic and two-factor interaction effects while ensuring that all design points within the biologically safe range. Coding the factors at −1 (pH 6, 10 °C, 10 psu), 0 (pH 8, 20 °C, 35 psu), and +1 (pH 10, 30 °C, 60 psu), the FCCCD provides a practical balance between experimental coverage and feasibility. In contrast, a rotatable CCD would require an α value of approximately 1.68 for three factors—based on the formula α = (2^k^)^¼^, where *k* is the number of factors. This would push at least one factor beyond the safe operational range. A BBD, while more economical in terms of run number, does not include the corner points necessary for adequately capturing curvature at the extremes of the design space.

As shown above, ChatGPT explained that FCCCD is the appropriate design because it allows accurate estimation of quadratic and two-factor interaction effects while keeping all runs within the biologically safe range. In contrast, a rotatable CCD (α ≈ 1.68 for three factors) would extend axial points beyond the safe limits, and a BBD lacks corner points needed to capture curvature at the edges of the design space. Based on ChatGPT’s recommendation, a FCCCD (α = 1) was applied. Axial points were positioned on the faces of the design cube (coded ±1), ensuring that all experimental conditions remained within the biologically relevant range (pH 6, 8, or 10; 10, 20, or 30 °C; and 10, 35, or 60 psu) (Table 1). The design included eight factorial points, six axial points, and six center points, and each point was run in duplicate for a total of 40 runs. The replicated center points enabled the estimation of pure error and reliable assessment of variability under identical conditions (Table 2).

After performing algal growth tests according to the DoE, the quadratic model showed good agreement with the experimental data, with R^2^ = 88.35% and adjusted R^2^ = 83.78% (Table 3).

The overall model *F*-value was 19.31 (*p* < 0.001), confirming a significant fit. Among the linear terms, pH and temperature were significant (*p* < 0.05), whereas salinity was not (*p* = 0.889). Quadratic terms had the greatest influence (*F* = 60.97, *p* < 0.001), and the quadratic effects of pH, salinity, and temperature were all significant in that order. Salinity was insignificant in its linear form but significant in its quadratic form (*F* = 11.51, *p* = 0.002). According to the Pareto chart of the standardized effects, analysis showed that the quadratic pH term was the dominant effect, followed by linear pH, linear temperature, and quadratic salinity. Residual plots confirmed homoscedasticity and an approximately normal error distribution, with no systematic patterns related to time or fitted values. These results support the adequacy of the model (Supplementary Figures S1 and S2).

**Figure 2:**
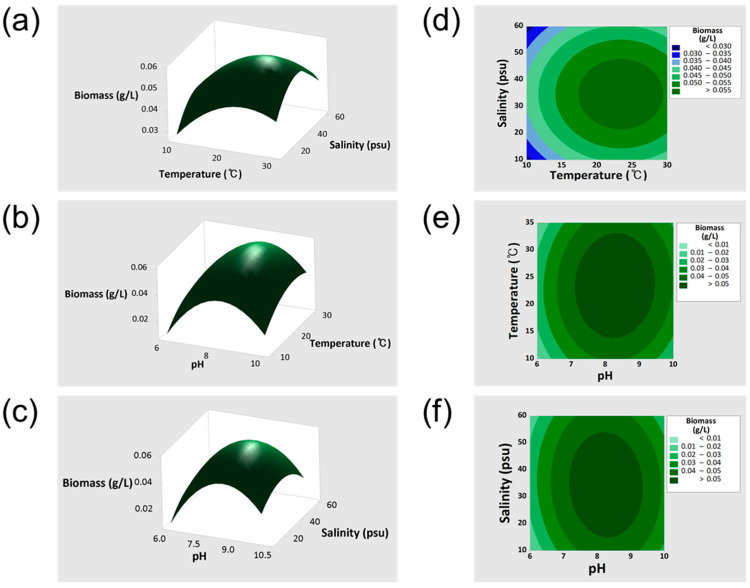
Three-dimensional response surface and contour plots for biomass production of *Gedaniella flavovirens* GFTA21 generated by response surface methodology (RSM). (**a**) 3D surface plot showing the interaction between temperature and salinity; (**b**) the interaction between temperature and pH; (**c**) the interaction between pH and salinity; (**d**) contour plot of temperature and salinity; (**e**) contour plot of temperature and pH; and (**f**) contour plot of pH and salinity.

The 3D response surface and contour plots represent biomass production (g/L) as a function of temperature (°C), salinity (psu), and pH (Figure 2a–f). Biomass production increased under specific combinations of these factors, with notable effects from temperature, salinity, and pH, as well as their interactions, particularly temperature–pH and salinity–pH. Biomass production (g/L) from 40 experimental runs were used to fit the quadratic response surface model, as shown in Equation (2).

Biomass (g/L) = −0.4705 + 0.1090·*A* + 0.00407·*B* + 0.001763·*C* − 0.006592·*A*^2^ − 0.000099·*B*^2^ − 0.000021·*C*^2^ + 0.000072·*A*·*B* − 0.000040·*A*·*C* (2)

Where *A*, *B*, and *C* represents pH, temperature (°C), and salinity (psu), respectively.

**Figure 3:**
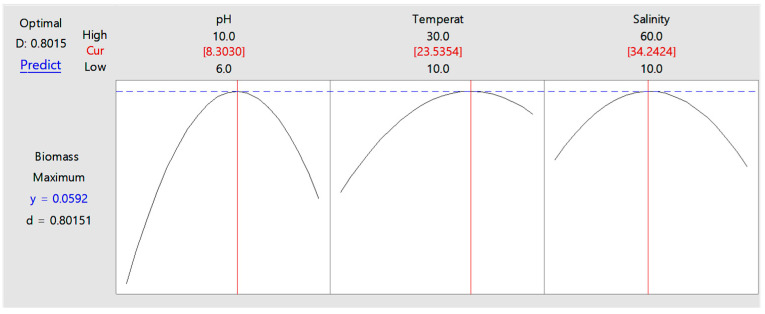
Response optimizer plot generated using Minitab software for *Gedaniella flavovirens* GFTA21, showing the predicted conditions for maximum biomass production. Vertical red lines indicate the predicted optimal value for each factor, and blue dashed lines represent the upper confidence boundaries estimated by the optimizer. The optimal parameters were pH 8.30, temperature 23.54 °C, and salinity 34.24 psu, with a predicted biomass yield of 0.0592 g/L.

For diatom *G. flavovirens* GFTA21, predictions based on the maximum biomass production value obtained via the Response optimizer indicated that the optimal culture conditions for maximal biomass production were pH 8.30, a temperature of 23.54 °C, and salinity of 34.24 psu (Figure 3).

In comparison, *G. panicellus* GPYS21, a species from the same genus, previously exhibited optimal growth at pH 7.33, 20.50 °C, and 42.32 psu based on CCD-based RSM analysis [27]. These results suggest that *G. flavovirens* prefers slightly higher pH and temperature than *G. panicellus* but requires lower salinity, reflecting possible species-specific environmental adaptations. Temperature, pH, and salinity are key environmental factors regulating diatom growth through effects on enzyme activity, osmotic balance, and photosynthetic efficiency [28,29,30,31]. Temperature influences enzymatic reactions and lipid metabolism by regulating acetyl-CoA-associated pathways, including branched-chain amino acid metabolism [32]. At low temperatures, membrane fluidity is maintained by increasing the proportion of unsaturated fatty acids [33], whereas higher temperatures reduce the synthesis of ribulose-1,5-bisphosphate carboxylase/oxygenase, a key enzyme in photosynthesis, thereby limiting carbon assimilation [34]. Elevated temperatures have also been reported to decrease the growth rate of thermo-intolerant marine diatoms, leading to reduced photosynthetic efficiency and metabolic imbalance [35]. pH alters the equilibrium between CO_2_ and HCO_3_^−^, directly affecting carbon fixation and ATP generation via proton gradients [36]. Salinity regulates intracellular ion homeostasis, and extreme salinity induces oxidative stress and impairs cellular functions [37,38].

**Figure 4:**
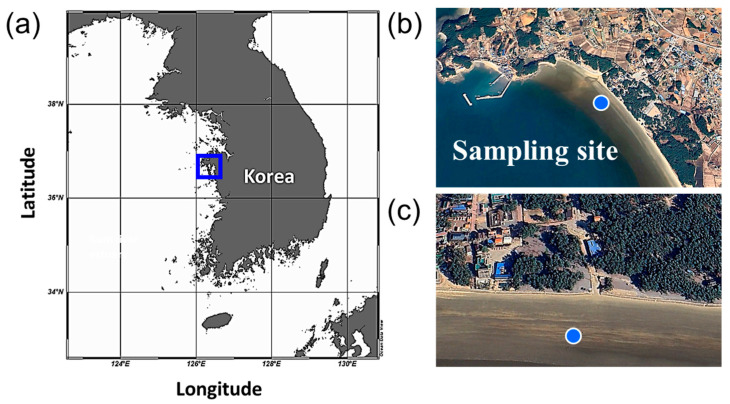
Sampling site of *Gedaniella flavovirens* GFTA21 from brackish waters in South Korea. (**a**) Map of the sampling site in Taean-gun, Chungcheongnam-do, with the target region indicated by a blue box. (**b**) Closer view of the coastal environment. (**c**) Detailed view showing the exact sampling point (blue dot). Satellite images were obtained from Google Earth.

In our study, biomass production increased toward moderately alkaline and mesothermal conditions (pH 8–9, 20–25 °C), likely reflecting enhanced activity of carbon fixation enzymes such as Rubisco and improved energy conversion efficiency within photosystems. Conversely, growth suppression under lower pH and temperature could be attributed to reduced CO_2_ availability and decreased membrane fluidity, limiting nutrient transport. The negligible effect of salinity on growth suggests efficient ionic regulation by compatible solutes (e.g., proline, betaine) and membrane-bound ion pumps, which maintain osmotic and redox balance under fluctuating salt conditions [37,38]. Such physiological plasticity is consistent with the euryhaline lifestyle of *G. flavovirens* and distinguishes it from its congener *G. panicellus*, which exhibits narrower tolerance and lower adaptability to salinity shifts. For example, *P. tricornutum* grows optimally at 30 ‰, with deviations leading to growth inhibition and decreased pigment concentrations [39]. *G. flavovirens* has been reported as an euryhaline diatom inhabiting brackish environments [19,20]. In this study, salinity did not significantly affect biomass (*F* = 0.02, *p* = 0.889), likely reflecting the broad tolerance range of the strain. The GFTA21 strain was isolated from a coastal area with substantial freshwater inflow, consistent with its adaptation to fluctuating salinity levels (Figure 4). Similarly, *Thalassiosira pseudonana*, a euryhaline and eurythermal diatom isolated from ballast water, exhibited optimal growth at 20–25 °C and 10–25 psu, while maintaining viability across a broad salinity range (0–30 psu) [40]. This adaptive flexibility parallels the physiological characteristics observed in *G. flavovirens* in our study, suggesting that tolerance to fluctuating salinity and moderate temperatures may be a common ecological strategy among coastal diatoms inhabiting dynamic estuarine environments.

Previous studies have shown that RSM is a robust and efficient tool for optimizing microalgal biotechnology processes, including culture conditions, flocculation efficiency, and medium composition [41,42,43]. Kirrolia et al. [40] used BBD to optimize *Chlorella* spp. culture by adjusting nitrate, phosphate, glucose, and pH, resulting in improved growth and lipid accumulation. Akış et al. [42] applied CCD to optimize pH-induced flocculation in marine and freshwater microalgae, achieving 92.63% flocculation efficiency for *Nannochloropsis oculata* (Droop) D.J.Hibberd 1981 at pH 10.5. Fawzy and Alharthi [43] optimized the medium composition of *Dunaliella parva* W.Lerche 1937 to enhance biodiesel production. Together, these studies demonstrate the potential of RSM for improving algal growth and biotechnological processes.

This study is the first to integrate ChatGPT-4.0, a large language model (LLM), with RSM modeling to optimize *G*. *flavovirens* GFTA21 culture conditions. Although RSM is efficient, defining appropriate design can be challenging. ChatGPT-4.0 addressed this limitation by guiding the experimental design with predictive logic and contextual understanding.

### 3.2. Results of Fatty Acid and Photosynthetic Pigment Analysis

To evaluate the industrial applicability of the newly isolated *G. flavovirens* GFTA21 strain, its cellular components, including fatty acids and photosynthetic pigments, were analyzed. The total fatty acid content was 200.10 mg/g (Table 4), with palmitoleic acid (C16:1) as the dominant component (116.61 mg/g; 58.28%). Palmitic acid (C16:0) was the second most abundant (37.82 mg/g; 18.90%), followed by eicosapentaenoic acid (EPA, C20:5) (22.60 mg/g; 11.29%) and arachidonic acid (ARA, C20:4) (12.43 mg/g; 6.21%). Minor fatty acids included myristic acid (C14:0), stearic acid (C18:0), oleic acid (C18:1), linoleic acid (C18:2), gamma-linolenic acid (GLA, C18:3), and dihomo-gamma-linolenic acid (DGLA, C20:3).

With increasing demand for biofuel feedstocks, food products, and animal feed, lipid-accumulating microalgae are gaining commercial interest [44]. Identifying species that produce valuable fatty acids is therefore important. According to Cho et al. [27], *G. panicellus* GPYS21, a species in the same genus as GFTA21, has a total fatty acid content of 114.86 mg/g, with palmitoleic acid (C16:1) as the dominant component (71.64 mg/g, 62.37%), followed by palmitic acid (C16:0) (30.69 mg/g, 26.72%), EPA (C20:5) (7.92 mg/g, 6.90%), and myristic acid (C14:0) (1.84 mg/g, 1.60%). While both *G. panicellus* and *G. flavovirens* primarily produce palmitoleic and palmitic acids, species-specific differences are evident. *G. flavovirens* GFTA21 has a higher total fatty acid content (200.10 mg/g), about 1.74 times that of *G. panicellus* GPYS21 (114.86 mg/g). Although GFTA21 has a slightly lower proportion of palmitoleic acid (58.28% vs. 62.37%), its absolute yield is higher (116.61 mg/g vs. 71.64 mg/g). EPA content is also significantly greater in GFTA21 (22.60 mg/g, 11.29%) than in GPYS21 (7.92 mg/g, 6.90%). These results indicate that *G. flavovirens* is a promising industrial resource for palmitoleic acid and EPA production, and further research to enhance these components could improve its industrial potential.

In saturated fatty acids, C16 and C18 are primarily synthesized in plastids via the fatty acid synthase pathway, with elongation, desaturation, and triacylglycerol assembly occurring in the endoplasmic reticulum [45]. Their proportions vary by species, with some microalgae favoring C16 over C18 [46]. Diatoms predominantly accumulate C16 fatty acids while producing limited C18 variants [9]. For example, *Chaetoceros* sp. contains 55.2% C16 and only 5.0% C18 fatty acids [47], whereas *Nannochloropsis* sp. (Ochrophyta) contains 47.3% C16 and 10.1% C18 [48].

Palmitoleic acid has diverse bioactivities, including skin-whitening and antibacterial effects, making it suitable for cosmetic applications. Yoon et al. [49] reported that it suppresses the expression of tyrosinase, tyrosinase-related proteins, and microphthalmia-associated transcription factors, suggesting potential as a skin-whitening agent. Watanabe et al. [50] showed that it selectively inhibits *Staphylococcus aureus* proliferation in emulsions and formulations. Palmitoleic acid also regulates metabolism in adipose tissue, the cardiovascular system, liver, muscle, and pancreas [27,51]. The global market for palmitoleic acid was valued at US $51.75 million in 2023 and is projected to reach US $80.87 million by 2030 [27].

EPA is widely recognized for cardiovascular protection, anti-inflammatory activity, and mental health benefits [52]. It lowers blood triglycerides, regulates blood pressure, and prevents platelet aggregation, potentially reducing cardiovascular risk. EPA also helps prevent arrhythmias, serves as a precursor to prostaglandins and leukotrienes, and reduces inflammation. In addition, it may support mental health by regulating plasma and serum cholesterol levels, which are associated with depression and suicide [52,53].

**Figure 5:**
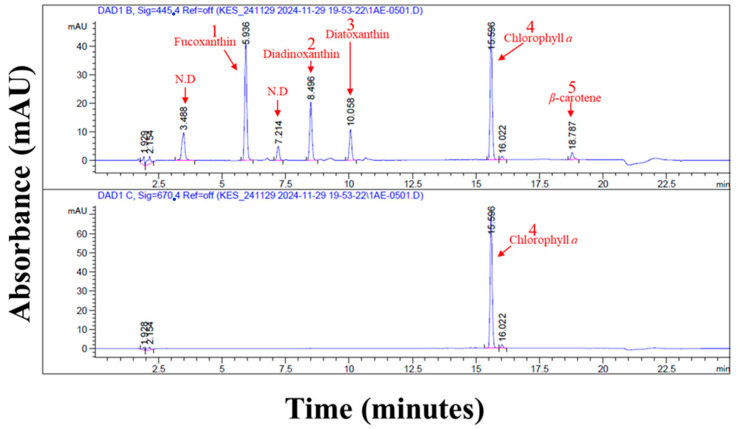
High-performance liquid chromatography (HPLC) equipped with a diode array detector (DAD) was used to analyze the photosynthetic pigments of *Gedaniella flavoviens* GFTA21. The upper panel (445 nm) and lower panel (670 nm) show absorption peaks corresponding to diadinoxanthin (2), diatoxanthin (3), chlorophyll *a* (4), and *β*-carotene (5), respectively. N.D. indicates a detected peak whose compound identity could not be determined.

As shown in Figure 5 and Table 5, *G. flavovirens* GFTA21 contained chlorophyll *a* (56.56 ± 1.62 mg/g), fucoxanthin (8.67 mg/g), diadinoxanthin (3.47 mg/g), diatoxanthin (2.16 mg/g), and *β*-carotene (0.46 mg/g). All pigments were identified by comparison with diode array detector (DAD) spectral scanning data (Supplementary Figure S3). Among these, chlorophyll *a* was the most abundant, followed by fucoxanthin, which was present at notably high levels.

According to Cho et al. [27], photosynthetic pigment analysis of *G. panicellus* GPYS21 revealed fucoxanthin (9.21 ± 0.68 mg/g), diadinoxanthin (2.21 ± 0.16 mg/g), chlorophyll *a* (22.36 ± 1.85 mg/g), and *β*-carotene (0.49 ± 0.03 mg/g). *G. flavovirens* GFTA21 had slightly lower fucoxanthin levels (8.67 ± 0.20 mg/g) than GPYS21 but still produced more fucoxanthin than most macro- and microalgae, indicating strong industrial potential [27]. For example, the macroalgae *Sargassum duplicatum* Bory 1828 (1.01 mg/g) and *Undaria pinnatifida* (Harvey) Suringar 1873 (0.73 mg/g) contain much lower fucoxanthin levels. Among microalgae, the diatoms *Chaetoceros calcitrans* (Paulsen) H.Takano 1968, *Cylindrotheca closterium* (Ehrenberg) Reimann & J.C.Lewin 1964, and *Cyclotella meneghiniana* Kützing 1844 produce 5.33 mg/g, 5.23 mg/g, and 2.30 mg/g, respectively. In contrast, well-known fucoxanthin producers such as *P. tricornutum* (16.51 mg/g) and *O. aurita* (23.30 mg/g) exceed GFTA21 in fucoxanthin content [27].

Most microalgae contain two major classes of pigments, chlorophylls and carotenoids, whereas cyanobacteria and some red microalgae also possess phycobiliproteins as part of their light-harvesting apparatus. Chlorophylls primarily function in capturing light energy through complex light-harvesting systems and converting it into chemical energy via the photosynthetic electron transport chain. Carotenoids act as accessory pigments that expand the light absorption spectrum and transfer captured energy to chlorophyll for photosynthesis. They also play a critical role in photoprotection, shielding cells from damage caused by excess light and quenching light-induced reactive oxygen species [54]. Carotenoids are classified into carotenes, xanthophylls, and oxygenated derivatives. In diatoms, *β*-carotene belongs to the carotenes, whereas fucoxanthin, diadinoxanthin, and diatoxanthin are classified as xanthophylls.

Among carotenoids, fucoxanthin is a key pigment in diatoms, absorbing light in the blue–green region and transferring the captured energy to the fucoxanthin–chlorophyll protein complex [55]. Although GFTA21 contained less fucoxanthin than *P. tricornutum* and *O. aurita*, previous studies suggest that optimization strategies such as oxidative stress induction or nutrient adjustment could enhance its accumulation [56,57]. These findings indicate that *G. flavovirens* GFTA21 represents a promising bioresource for commercial fucoxanthin production. Owing to its diverse bioactivities, fucoxanthin has broad industrial applicability, including aquaculture feed, functional foods, cosmetics, and the development of emulsions, nanoparticles, and hydrogels [58,59,60].

Budiarso et al. [61] comprehensively reviewed recent progress in microalgae-based fucoxanthin production, focusing on advanced cultivation strategies, eco-friendly extraction techniques, and molecular approaches such as CRISPR/Cas9 and synthetic biology to enhance biosynthesis. They emphasized the integration of cultivation optimization, metabolic engineering, and sustainable downstream processing to achieve scalable and cost-effective fucoxanthin production for nutraceutical, pharmaceutical, and cosmetic applications. In parallel, Chen et al. [62] reviewed the neuroprotective functions of fucoxanthin, describing its preventive and therapeutic potential against neurological disorders through multiple mechanisms, including antioxidation, anti-apoptosis, autophagy activation, and suppression of neuroinflammation.

The use of fucoxanthin-rich microalgae as aquaculture feed ingredients has been well documented. Ribeiro et al. [6] reported that supplementing gilthead seabream diets with the fucoxanthin-rich microalga *P. tricornutum* enhanced the characteristic golden-yellow pigmentation of the fish. Likewise, replacing 6% of fishmeal with *P. tricornutum* biomass in Atlantic salmon (*Salmo salar* Linnaeus, 1758) feed had no adverse effects on growth performance or proximate composition, including protein, lipid, energy, and ash content [63].

These findings suggest the potential of fucoxanthin-producing microalgae as natural pigments and functional ingredients in aquaculture feeds. Given the increasing demand for fucoxanthin-based products, the global fucoxanthin market was valued at approximately USD 2.1 billion in 2023 and is projected to reach USD 3.8 billion by 2031 [27].

### 3.3. ChatGPT-4.0-Based Evaluation of the Industrial Applicability of G. flavovirens GFTA21

To evaluate the commercial potential of *G*. *flavovirens* GFTA21, analyzed data on its photosynthetic pigments and fatty acid composition (Table 4 and Table 5) were provided to ChatGPT-4.0 for prompt-based assistance. Using this biochemical input, ChatGPT-4.0 prioritized potential industrial applications by relevance and feasibility. The following user–AI interaction illustrates the prompt-based prioritization process derived from the biochemical data.

User prompt: Please suggest potential industries for application based on the analyzed photosynthetic pigments and fatty acid components—such as nutraceuticals, pharmaceuticals, cosmetics, aquaculture, bioenergy, biopolymer, biomaterials, and others—and present your findings in a table, ranked by priority with corresponding justifications.

ChatGPT-4.0 response: The AI ranked aquafeeds as the most promising sector, based on the presence of key compounds such as EPA, ARA, and fucoxanthin, which are known to enhance pigmentation and growth in aquatic organisms. The ranking (Table 6) was guided by criteria including (i) compound abundance and known bioactivity, (ii) relevance to industry needs (e.g., pigmentation and immune enhancement in aquaculture), and (iii) feasibility of application based on existing literature.

ChatGPT ranked potential industries (e.g., aquafeed, nutraceuticals, cosmetics) by integrating the relative abundance of the identified pigments (fucoxanthin, *β*-carotene, chlorophyll *a*) and fatty acids (EPA, ARA, palmitoleic acid) with their reported bioactivities and market relevance. Specifically, the ranking considered three main aspects: (i) biological functionality of compounds (e.g., pigmentation, immune enhancement, cardiovascular benefits), (ii) commercial feasibility and current market demand, and (iii) consistency with established microalgal product applications. Each ranking outcome was subsequently reviewed and cross-validated with peer-reviewed studies on the utilization of similar compounds in related industrial sectors to ensure both scientific and economic plausibility.

As shown in Table 6, among the assessed industries, aquafeeds ranked first. This was attributed to the combined effects of fatty acids and pigments that support fish growth, enhance pigmentation, and improve aquaculture product quality. The nutraceutical industry ranked second, driven by the high levels of EPA and fucoxanthin, which provide cardiovascular, anti-inflammatory, and anti-obesity benefits, making them valuable for health supplements. Functional foods ranked third, as *G. flavovirens* GFTA21 contains abundant EPA, ARA, and carotenoids that enhance the nutritional value of food products and align with growing health-conscious consumer trends.

The cosmetic industry was placed fourth, supported by the presence of fucoxanthin, *β*-carotene, and chlorophyll *a*, which offer antioxidant, anti-aging, and skin-brightening effects suitable for premium skincare formulations. The pharmaceutical industry ranked fifth, as EPA and ARA show therapeutic potential for managing inflammation, metabolic disorders, and cardiovascular diseases, highlighting their value in advanced medical applications. Finally, the food colorant industry ranked sixth, supported by natural pigments such as *β*-carotene and fucoxanthin, which meet the rising demand for clean-label, health-promoting food colorants.

Based on the results of industrial applicability, the functional roles of the analyzed fatty acids and photosynthetic pigments were evaluated for potential applications in aquaculture feeds. Relevant literature was reviewed to identify supporting studies, including microalgal sources, feeding conditions, and target species, as summarized in Table 7.

Fucoxanthin was identified as a key pigment that enhances skin and fillet pigmentation and supports fish growth and nutrient retention, as demonstrated in feeding trials using *P. tricornutum* at inclusion levels of 2.5–6% in diets for gilthead seabream and Atlantic salmon [6,63]. *β*-carotene has been associated with growth promotion and immune enhancement in crustacean species such as Pacific white shrimp and black-tiger prawn, with dietary supplementation of *Dunaliella* species at 1–2% showing beneficial effects [64]. Among the fatty acids, EPA is an essential nutrient for marine fish larvae, improving growth, feed efficiency and muscle-lipid composition. Marine microalgae including *Nannochloropsis*, *Phaeodactylum*, *Isochrysis*, and *Chaetoceros* are widely used as EPA sources in aquaculture feeds and live-feed production [65]. Similarly, ARA improves larval survival and stress resilience, as shown in guppies when *Parietochloris incisa* (H.Reisigl) Shin Watanabe 1996 was used as a dietary supplement [66]. In addition, chlorophyll *a* exhibited antioxidant and DNA-protective effects in catfish (*Rhamdia quelen* (Quoy & Gaimard, 1824)) when residual biomass from *Acutodesmus obliquus* (Turpin) Hegewald & Hanagata 2000 containing chlorophylls was included at 1–3% in the diet. Fish fed with this biomass showed increased antioxidant enzyme activities and reduced DNA damage, indicating potential health benefits of chlorophyll-based compounds in aquaculture feeds [67]. Collectively, these findings suggest that *G. flavovirens* GFTA21, with its diverse bioactive compounds, is a promising source of functional ingredients for aquaculture feed formulations.

However, this AI-assisted evaluation was based solely on pigment and fatty acid profiles, which may not fully capture the overall biochemical potential of the strain. Other key metabolites, such as proteins, polysaccharides, and antioxidant molecules, may also play important roles in determining industrial suitability but were not included in this model. Future studies integrating multi-omics data and experimental validation will help refine the predictive accuracy of such AI-assisted analyses.

AI-based predictions are increasingly being applied in biotechnological research. For example, Zhu [68] proposed MetaPredictor, a deep learning-based in silico system incorporating AI assistance for drug metabolite prediction. Trained on large-scale datasets, MetaPredictor outperformed traditional rule-based and data-driven models, showing higher accuracy in predicting metabolic reactions and identifying novel metabolic pathways. AI integration improved prediction flexibility and precision while reducing time and cost in drug development. Maharjan et al. [69] reported that AI assistance significantly improves the performance of open-source LLMs in medical question–answering systems. This approach offers a clear advantage over conventional fine-tuning methods, as it generates highly accurate medical responses without requiring additional retraining. Given the sensitivity and limited accessibility of medical data, AI assistance reduces data requirements and enables rapid, efficient deployment. It also enhances the adaptability of LLMs across diverse medical domains, making AI-driven tools more practical for supporting healthcare professionals in diagnosis and treatment planning.

In addition, Tong and Zhang [18] demonstrated that ChatGPT can effectively summarize, interpret, and forecast research topics in synthetic biology through prompt-based dialogue, revealing its potential as a supplementary tool for scientific exploration. However, they also noted that the model’s accuracy and analytical depth remain limited, underscoring the necessity of careful human verification when applying AI-generated outputs in research. Similarly, Cahan and Treutlein [70] showed through an interactive dialogue with ChatGPT that computational and systems biology have substantially advanced stem cell research by enabling large-scale data integration, predictive modeling, and optimization of differentiation processes. Yet, they emphasized that AI-generated insights must be grounded in explicit theoretical frameworks to extract causal biological understanding.

As AI-based predictive models are being increasingly utilized, their applications are expected to expand to metabolic network analysis, physiological response prediction, and gene expression regulation. These models are anticipated to play key roles in metabolic engineering and biorefinery applications, further advancing biotechnological innovation. However, limitations remain, particularly regarding reference mismatches and unvalidated information. Such limitations are largely due to copyright restrictions by academic publishers, which hinder large-scale data access and web crawling [16].

Despite these limitations, our findings indicate that ChatGPT-4.0 provides reasonable bioactivity predictions, although manual validation remains essential for accurate reference verification. This study revealed that the potential of ChatGPT-4.0-assisted bioactivity prediction as a valuable tool for evaluating the commercial applicability of algal strains.

Although challenges remain, this study showed AI-based approaches hold significant promise for algal strain optimization, bioactivity evaluation, and industrial feasibility assessment. Consequently, the ChatGPT-4.0-assisted approach can be an efficient and practical strategy for rapidly assessing the industrial potential of novel microalgal strains and guiding future research directions.

## 4. Conclusions

In this study, we optimized the culture conditions of *G*. *flavovirens* GFTA21 through RSM modeling with the assistance of ChatGPT-4.0. After cultivating the strain under the optimized conditions, we analyzed its fatty acid and photosynthetic pigment compositions. Based on these analytical results, we applied ChatGPT-4 to predict the industrial applicability of GFTA21, which identified aquaculture feeds as the top-ranked sector. To further explore this potential, we employed ChatGPT-4 to map the functional activities of the identified fatty acids and pigments in relation to aquaculture feeds, particularly their roles in fish growth, immune function, and pigmentation. These predicted bioactivities were subsequently verified through an extensive literature review. Collectively, the findings confirm the high potential of GFTA21 as a valuable bioresource for aquaculture feeds. This study demonstrates that combining ChatGPT-assisted RSM optimization with AI-based industrial prediction and functional analysis provides an effective strategy for rapidly evaluating the industrial potential of novel microalgal strains.

## 5. Patents

Cho, K.; An, S.M.; Hwang, H.-J.; Kim E.S.; Lee D.-S. Novel *Gedaniella flavovirens* GFTA21 strain and its optimal culture condition and use. Korean patent application No. 10-2025-0095792, filed on 16 July 2025.

## Figures and Tables

**Table 1:** Conversion of coded levels (−1, 0, +1) to actual pH, temperature (°C), and salinity (psu) used in the face-centered central composite design (FCCCD).

Parameters	Level		
	−1	0	1
pH	6	8	10
Temperature (°C)	10	20	30
Salinity (psu)	10	35	60

**Table 2:** Face-centered central composite design (FCCCD) matrix showing coded levels of pH, temperature (°C), and salinity (psu) with corresponding biomass responses. This matrix served as the experimental basis for response surface-guided biomass production optimization.

No.	pH	Temperature (°C)	Salinity (psu)	Biomass (g/L)
1	−1	1	1	0.01
2	1	−1	1	0.01
3	1	1	−1	0.04
4	−1	−1	−1	0.00
5	−1	−1	−1	0.01
6	−1	1	1	0.01
7	0	0	0	0.07
8	1	−1	1	0.01
9	0	0	0	0.07
10	1	1	−1	0.02
11	0	0	0	0.04
12	0	0	0	0.06
13	−1	−1	1	0.00
14	1	−1	−1	0.02
15	1	1	1	0.02
16	1	1	1	0.02
17	0	0	0	0.06
18	0	0	0	0.07
19	−1	−1	1	0.00
20	−1	1	−1	0.01
21	1	−1	−1	0.02
22	0	0	0	0.05
23	−1	1	−1	0.01
24	0	0	0	0.06
25	0	0	0	0.05
26	0	−1	0	0.02
27	0	0	0	0.06
28	0	0	1	0.03
29	−1	0	0	0.01
30	0	0	1	0.04
31	0	1	0	0.06
32	0	−1	0	0.02
33	1	0	0	0.02
34	1	0	0	0.03
35	0	0	−1	0.02
36	−1	0	0	0.01
37	0	1	0	0.05
38	0	0	−1	0.03
39	0	0	0	0.06
40	0	0	0	0.06

**Table 3:** Results of analysis of variance (ANOVA) for response surface quadratic model (RSM) by Minitab Statistical Software.

Source	DF	Adj. SS	Adj. MS	*F*	*p*
Model	11	0.016667	0.001515	19.31	<0.001
Linear	3	0.001904	0.000635	8.09	<0.001
pH (A)	1	0.000989	0.000989	12.61	0.001
Temperature (B)	1	0.000912	0.000912	11.63	0.002
Salinity (C)	1	0.000002	0.000002	0.02	0.889
Square	3	0.014352	0.004784	60.97	<0.001
A·A	1	0.003733	0.003733	47.57	<0.001
B·B	1	0.000526	0.000526	6.71	0.015
C·C	1	0.000903	0.000903	11.51	0.002
Interaction	3	0.000096	0.000032	0.41	0.748
A·B	1	0.000012	0.000012	0.42	0.524
A·C	1	0.000063	0.000063	0.81	0.377
B·C	1	<0.001	<0.001	0.00	0.988
Error	28	0.002197	0.000078		
Lack-of-Fit	5	0.001226	0.000245	5.81	0.001
Pure Error	23	0.000971	0.000042		
Total	39	0.018864			

R^2^ = 88.35%, adj. R^2^ = 83.78%. DF, degrees of freedom; Adj. SS, adjusted sum of squares; Adj. MS, adjusted mean of squares.

**Table 4:** Fatty acid composition of diatom *Gedaniella flavovirens* GFTA21 extract analyzed by gas chromatography–mass spectrometry (GC/MS). Values are presented as mean ± standard deviation (SD).

Fatty Acid		Category	Amount (mg/g)	Weight (*w*/*w*. %)
Myristic acid	C14:0		3.40 ± 0.00	1.70
Palmitic acid	C16:0		37.82 ± 0.02	18.90
Palmitoleic acid	C16:1	ω-7	116.61 ± 0.04	58.28
Stearic acid	C18:0		0.42 ± 0.00	0.21
Oleic acid	C18:1	ω-9	1.64 ± 0.00	0.82
Linoleic acid	C18:2	ω-6	3.04 ± 0.00	1.52
Gamma-linolenic acid	C18:3	ω-6	1.60 ± 0.00	0.80
Dihomo-gamma-linolenic acid	C20:3	ω-6	0.53 ± 0.00	0.27
Arachidonic acid	C20:4	ω-6	12.43 ± 0.03	6.21
Eicosapentaenoic acid	C20:5	ω-3	22.60 ± 0.04	11.29
Total fatty acids			200.10	100.00

**Table 5:** Photosynthetic pigment composition of diatom *Gedaniella flavovirens* GFTA21 analyzed using high-performance liquid chromatography with diode array detection (HPLC-DAD). Values are presented as mean ± standard deviation (SD).

Pigments	Retention Time (min)	Peak Area	Amount (mg/g)
Fucoxanthin	5.94 ± 0.00	249.27 ± 11.55	8.67 ± 0.20
Diadinoxanthin	8.50 ± 0.00	129.37 ± 5.67	3.47 ± 0.07
Diatoxanthin	10.06 ± 0.00	69.41 ± 3.09	2.16 ± 0.05
Chlorophyll *a*	15.59 ± 0.01	412.99 ± 1.52	56.56 ± 1.62
*β*-carotene	18.78 ± 0.01	18.05 ± 0.85	0.46 ± 0.01

**Table 6:** Estimated ranking of industrial applicability and rationale for the potential of the diatom *Gedaniella flavovirens* GFTA21, as evaluated by ChatGPT-4 based on its bioactivities and cellular bioactive components.

Rank	Industry	Reason for Potential
1	Aquafeeds	Fatty acids and pigments support fish growth and improve pigmentation in aquafeeds, enhancing product quality for the aquaculture industry.
2	Nutraceuticals	High content of Eicosapentaenoic acid (EPA) and fucoxanthin, offering cardiovascular, anti-inflammatory, and anti-obesity benefits. These compounds are highly valued in health supplements.
3	Functional Foods	Abundance of EPA, Arachidonic acid (ARA), and carotenoids, which enhance the nutritional profile of food products, supporting health-conscious consumer trends.
4	Cosmetics	Rich in fucoxanthin, *β*-carotene, and chlorophyll *a*, which provide antioxidant, anti-aging, and skin-brightening effects. Ideal for premium skincare products.
5	Pharmaceuticals	EPA and ARA have therapeutic properties for managing inflammation, metabolic disorders, and cardiovascular diseases, making them essential in advanced medicine.
6	Food Colorants	Natural pigments such as *β*-carotene and fucoxanthin meet the demand for clean-label, health-promoting food colorants in the food industry.

**Table 7:** Photosynthetic pigments and bioactive fatty acids identified in *Gedaniella flavovirens* GFTA21 and their potential benefits in aquaculture applications. Functional roles were first estimated using ChatGPT-4 and subsequently verified based on existing literature. Microalgal sources, feeding conditions, and target species from relevant studies are summarized.

Compounds (Class)	Aquaculture Benefit(s)	Microalgal Source/ Feeding Trial	References
Fucoxanthin (xanthophyll)	Skin and fillet pigmentation enhancement; supports growth and nutrient retention	*Phaeodactylum tricornutum*/whole biomass, 2.5–6% of diet (gilthead seabream, Atlantic salmon)	[6,63]
*β*-Carotene (carotene)	Growth promotion; immune enhancement	*Dunaliella* sp./1–2% algal meal in diet (Pacific white shrimp & black-tiger prawn)	[64]
EPA (20:5 n-3 PUFA)	Improved growth rate, feed efficiency, and muscle lipid composition	Various marine microalgae/commonly used in aquaculture feeds and live feed production for fish larvae and shellfish	[65]
ARA (20:4 n-6 PUFA)	Improved larval survival and resilience to stress	*Parietochloris incisa*/diet supplement during first-month fry stage (guppy)	[66]
Chlorophyll *a* (tetrapyrrole)	Enhanced antioxidant status and DNA protection	*Acutodesmus obliquus*/1–3% residual algal biomass in diet (*Rhamdia quelen*)	[67]

## Data Availability

The data presented in this study are available on request from the corresponding author.

## Supplementary Materials

The following supporting information can be downloaded at: https://www.mdpi.com/article/10.3390/bioengineering12111277/s1, Figure S1: Pareto chart showing the standardized effects of RSM factors on biomass of *Gedaniella flavovirens* GFTA21. The quadratic term of pH (AA) shows the largest effect on biomass, followed by the linear pH term (A), and temperature (B). Figure S2: Residual diagnostic plots for the RSM biomass model of *Gedaniella flavovirens* GFTA21, including checks for independence, homoscedasticity, and normality. Figure S3: Chromatograms obtained by high-performance liquid chromatography with diode-array detection (HPLC-DAD) for major pigments in *Gedaniella flavovirens* GFTA21, compared with corresponding standard spectra.

## Abbreviations

AI	Artificial Intelligence
DoE	Design of Experiments
EPA	Eicosapentaenoic Acid
FCCCD	Face-centered central composite design
LLM	Large Language Model
PSU	Practical Salinity Unit
RSM	Response Surface Methodology
