# Supplementary material for: AI-Assisted Response Surface Methodology for Growth Optimization and Industrial Applicability Evaluation of the Diatom Gedaniella flavovirens GFTA21
Source: Bioengineering (Basel). 2025 Nov 20;12(11):1277. doi: 10.3390/bioengineering12111277 (PMC12650378; doi:10.3390/bioengineering12111277)
Supplement: Supplementary file 1 [file bioengineering-12-01277-s001.zip › bioengineering-3953930-supplementary.pdf]

Supplementary Figure S1. Pareto chart showing the standardized effects of RSM factors on biomass of *Gedaniella flavovirens* GFTA21. The quadratic term of pH (AA) shows the largest effect on biomass, followed by the linear pH term (A), and temperature (B).

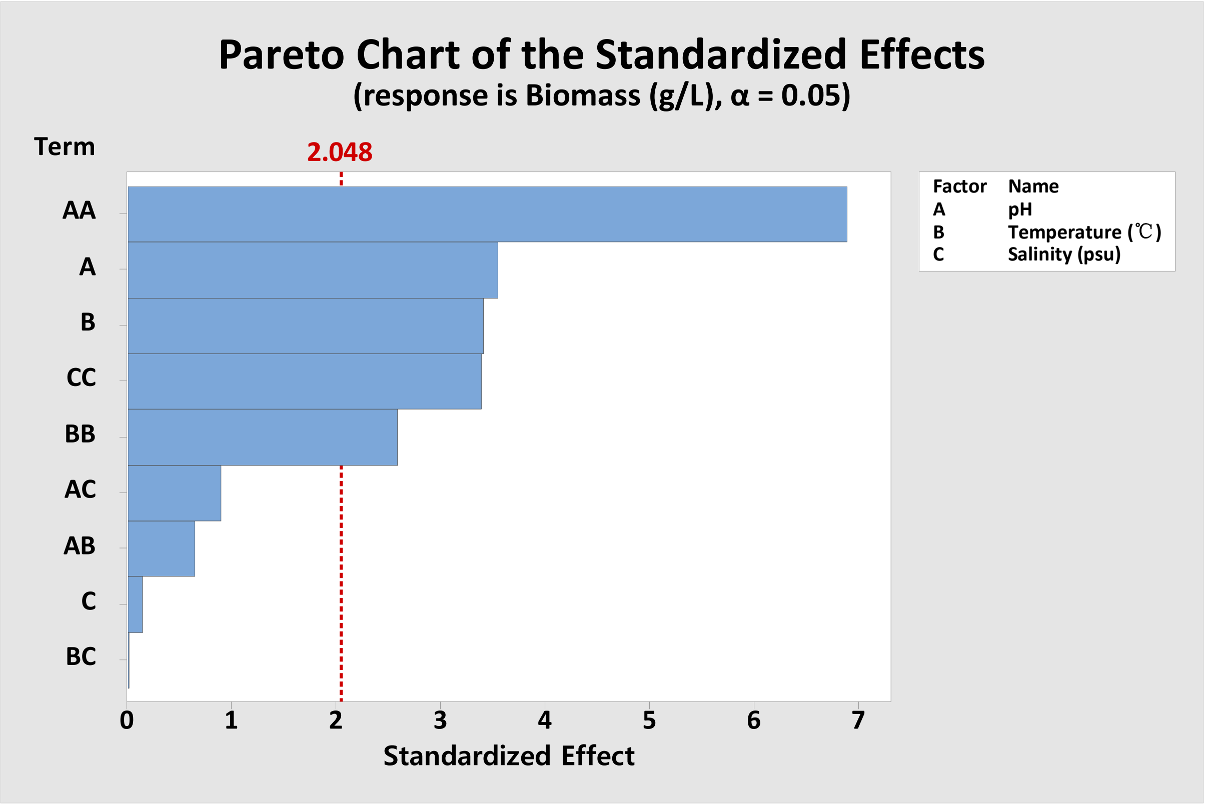

Supplementary Figure S2. Residual diagnostic plots for the RSM biomass model of *Gedaniella flavovirens* GFTA21, including checks for independence, homoscedasticity, and normality.

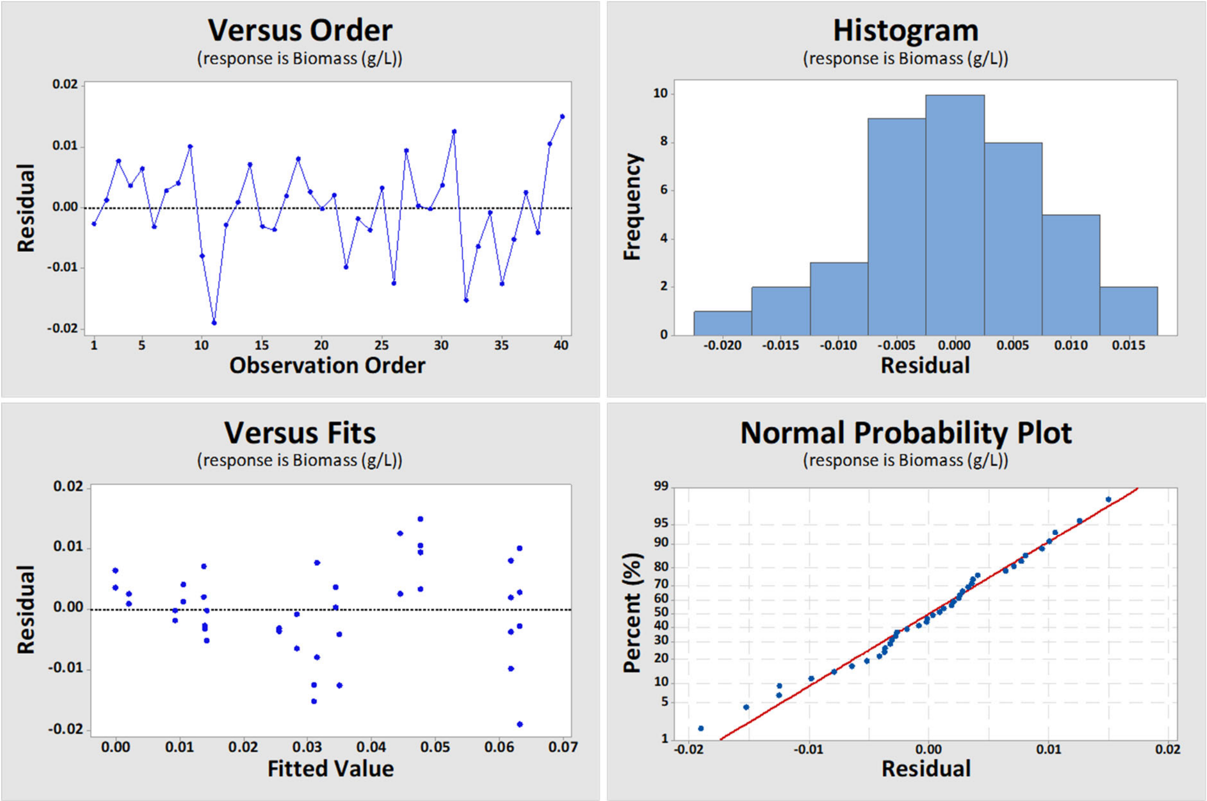

Supplementary Figure S3. Chromatograms obtained by high-performance liquid chromatography with diode-array detection (HPLC-DAD) for major pigments in Gedanienella flavovirens GFTA21, compared with corresponding standard spectra.

Pigment	Standard spectrum	GFTA21 spectrum
Fucoxanthin	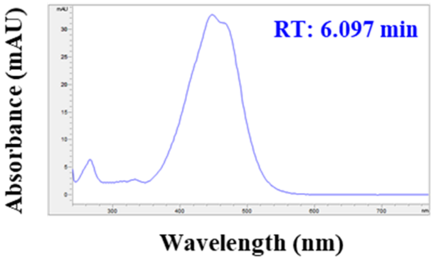	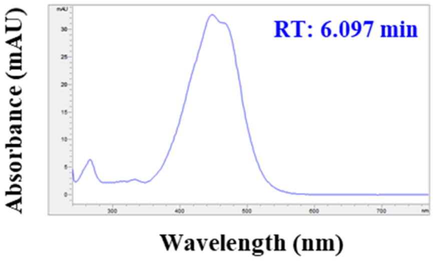
Diadinoxanthin	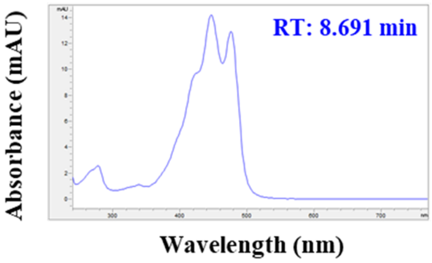	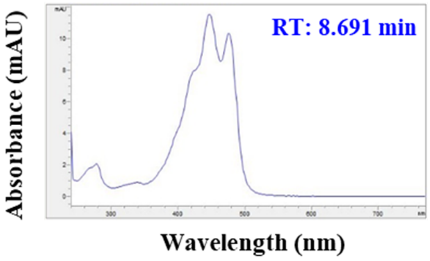
Diatoxanthin	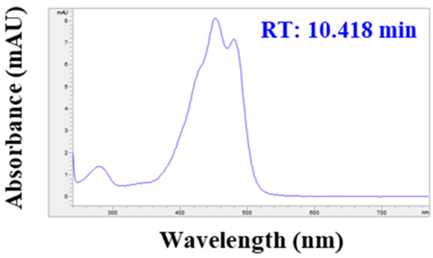	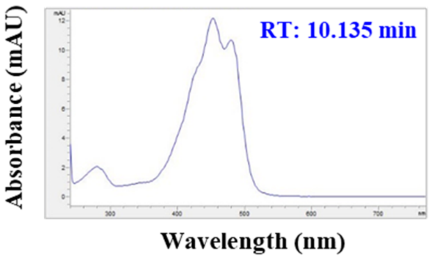
Chlorophyll *a*	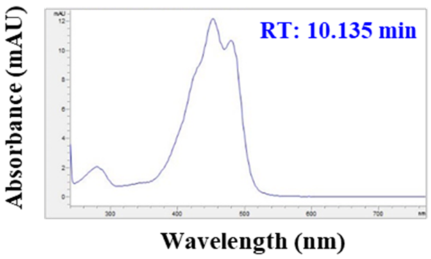	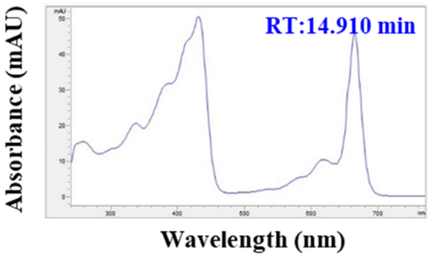
β-carotene	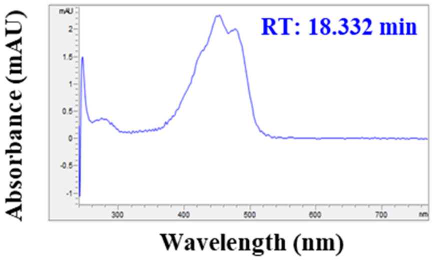	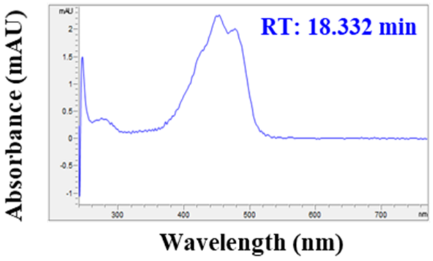
